# A Chemical Mechanistic Path Leads the Way to Cellular Argpyrimidine

**DOI:** 10.1021/jacs.5c09369

**Published:** 2025-10-10

**Authors:** Vo Tri Tin Pham, Suprama Datta, Amy C. Sterling, Stefan M. Hansel, Rebecca A. Scheck

**Affiliations:** Department of Chemistry, Tufts University, 62 Talbot Avenue, Medford, Massachusetts 02155, United States

## Abstract

Argpyrimidine (APY) is a methylglyoxal-derived advanced glycation end-product (AGE) that has been associated with multiple diseases. As APY forms without an enzyme, it remains exceptionally difficult to pinpoint where APY is likely to be found, both on individual proteins and in cells. In this study, we used a peptide model system and mass spectrometry analysis to investigate the chemical mechanism through which APY arises from methylglyoxal (MGO), a biologically relevant glycating agent. Consistent with other proposed APY formation mechanisms, our results identify AGE species with a mass change of [M + 144], presumably including tetrahydropyrimidine (THP), as a direct precursor to APY. However, our results rule out previously proposed reductone or oxidative decarboxylation mechanisms. Instead, we show that a formal oxidation step is not required, and that formate is released instead of CO_2_. We further show the potential for a nearby residue such as Tyr to assist in the APY formation mechanism by acting as a general base. These experiments also reveal that phosphorylated Tyr or Ser residues can also promote equivalent levels of APY formation, despite introducing additional negative charges that we previously showed to impede glycation. Guided by these mechanistic insights and a newly defined role for phosphorylated residues on glycation substrates, we performed quantitative bottom-up proteomics analysis for MGO-treated cells. Gene ontology and functional annotation clustering analyses for APY-modified proteins suggested a correlation with phosphorylation-related terms (e.g., kinase activity or protein phosphorylation), which was validated using synthetic phosphopeptide substrates. Collectively, these data define a chemical mechanistic path to APY and suggest significant crosstalk between cellular phosphorylation and glycation events including APY formation.

## Graphical abstract

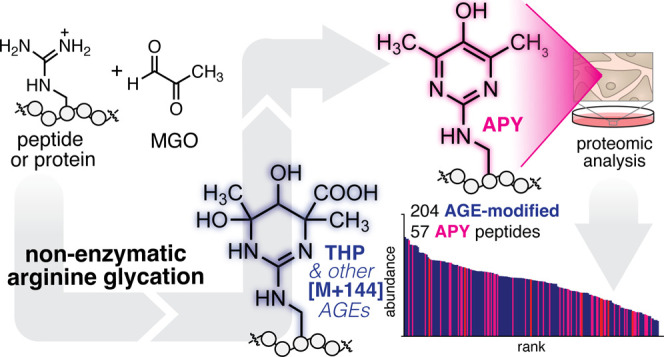

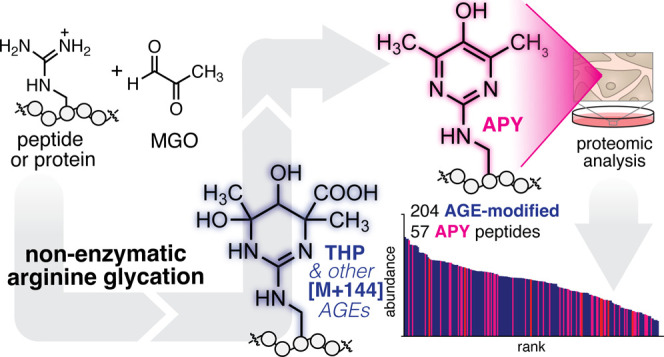

## Introduction

Argpyrimidine (APY) is one of a group of nearly 40 protein post-translational modifications (PTMs) known as advanced glycation end-products (AGEs).^1–3^ AGEs are hallmarks of aging and disease that form nonenzymatically on Lys, the N-terminus, or—in the case of APY—on Arg residues.^1–3^ Despite a relatively high p*K*_a_, the Arg guanidino group reacts selectively with α-oxoaldehydes such as the biological glycating agent methylglyoxal (MGO), forming multiple AGEs.^4–6^ In addition to APY, these include the methylglyoxal-derived hydroimidazolone isomers (MGH-1, -2, & −3), dihydroxyimidazolidine (MGH-DH), carboxyethylarginine (CEA), and tetrahydropyrimidine (THP) (Figure 1). Although there is accumulating evidence that certain AGEs, especially the MGH isomers and CEA, lead to specific biological consequences,^7–12^ far less is known about the biology of APY. Several reports have correlated APY with cellular processes like apoptosis, autophagy, or chaperone activity, and a few have shown its association with diseases like brunescent cataractous lens, filial amyloidotic polyneuropathy, or cancer.^13–21^ Still, there are virtually no causal connections that link APY to specific biological consequences.

**Figure 1:**
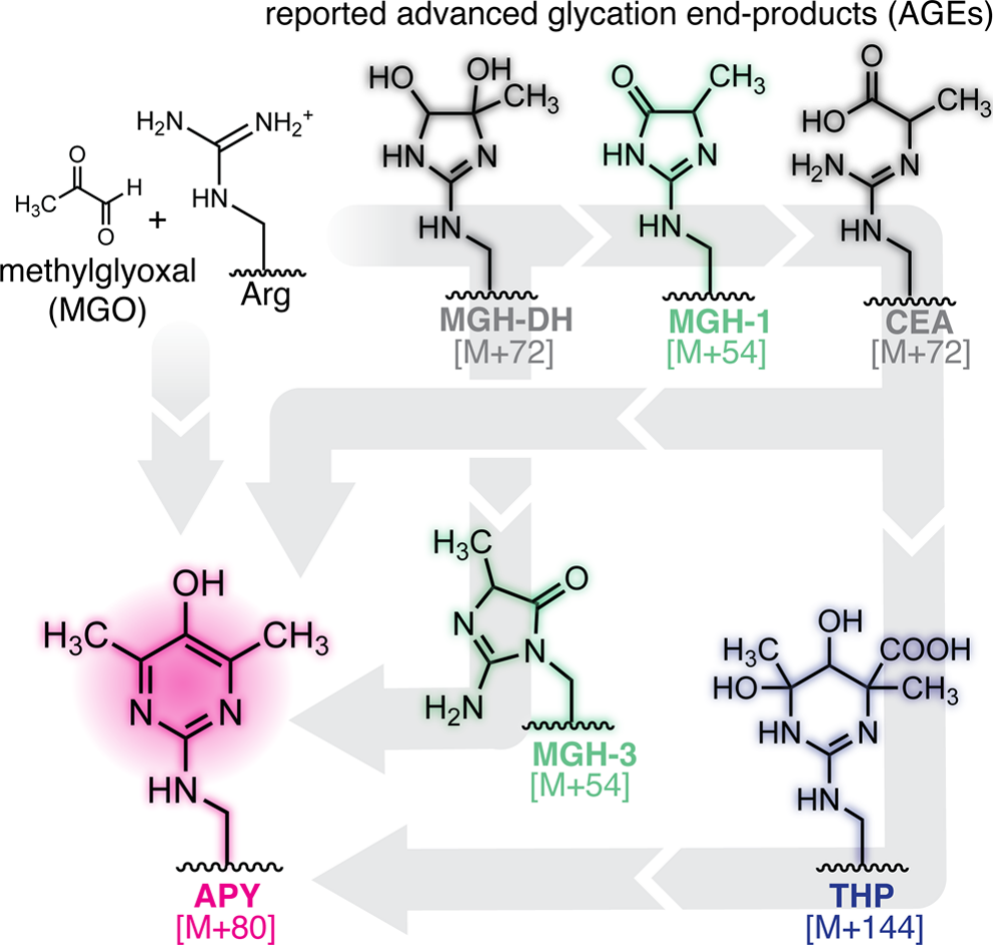
**Overview of MGO-derived advanced glycation end-products.** In addition to APY, these include the methylglyoxal-derived hydroimidazolone isomers (MGH-1, -2, & −3), dihydroxyimidazolidine (MGH-DH), carboxyethylarginine (CEA), and tetrahydropyrimidine (THP). Several possible pathways for APY formation have been previously proposed (gray arrows).

These open questions persist because APY has been notoriously difficult to track due to a lack of highly specific tools and an unknown mechanism of formation. Given that APY requires more than a single MGO molecule to form, it could be particularly relevant under hyperglycemic conditions. Additionally, it has been proposed that APY formation also depends on oxidative stress, suggesting that it could be a critical link between glycation and other cellular stresses.^20,22,23^ It is therefore essential to understand APY’s formation mechanism to pinpoint the features that influence its formation and the locations—both on proteins and in cells—where APY is more likely to accumulate.

Previous work in our lab has shown that specific Arg-derived AGEs depend not only on MGO concentrations, but also the surrounding chemical microenvironment.^4,5^ We have shown that several of these Arg-derived AGEs are mechanistically connected and can interconvert.^4^ In this work, we use a peptide model system to determine chemical features that favor APY formation. We show that although APY is generated more slowly than other AGEs, it is also quite stable once formed. We also confirm that [M + 144] species, presumably including tetrahydropyrimidine (THP), are a direct precursor to APY, and we propose a new APY formation mechanism that involves an initial retro-aldol step, ultimately generating formate as a byproduct, rather than an oxidative decarboxylation. We further demonstrate that the presence of a nearby Tyr can facilitate APY formation. These experiments also revealed that phosphorylated Tyr or Ser residues could also facilitate APY formation, despite adding substantial negative charges that we have previously shown to inhibit glycation.^3–5^ These results prompted us to conduct quantitative proteomics experiments investigating APY formation in live cells treated with MGO, enabling us to draw a previously unknown connection between AGE formation and phosphorylation in a cellular context. These findings provide essential mechanistic insights that are greatly needed for future studies that seek to develop chemical or fluorescent tools that can be used to predict, sense, and study glycation events in living cells.

## Results

### Determining the APY Precursor

Although APY requires two molecules of MGO (72.02 Da), its mass change ([M + 80]) does not clearly match with an obvious mechanism involving loss or gain of water, as is the case for other AGEs like MGHs, MGH-DH, or CEA.^3^ This has made it particularly challenging to define the APY formation mechanism.^2,24,25^ Prior work from our lab and others’ points to tetrahydropyrimidine (THP, [M + 144]) as a possible precursor to APY.^4,24,25^ Specifically, we found that by observing AGE distributions for up to 4 weeks of incubation with MGO, there was an inversely proportional relationship between APY and [M + 144] species over time.^4^ We therefore initiated this study with the same hit sequence from our prior study (Ac-LESRHYA, peptide **1**) that was selected from a one-bead one-compound peptide library for its ability to form MGH-1.^4^ We treated peptide **1** (1 mM) with two equivalents (2 mM) of MGO, expecting that APY levels would increase at higher MGO concentrations. However, little to no APY was observed (<3% APY), even after 72 h of treatment, despite total glycation nearing 100% after 48 h, as assessed by liquid chromatography–mass spectrometry (LC–MS) (Figure 2A,B). In addition to peptide **1**, we also evaluated two additional peptide sequences, both from lens crystallin, that we previously identified as APY-modified using MGO-treated proteins in vitro (Figure S1).^5^ Even when increasing MGO concentrations to 3 mM, there was little to no (<3%) APY after 48 h across all three peptides, signifying that APY formation is largely independent of initial MGO concentrations, despite requiring two MGO equivalents to form.

**Figure 2:**
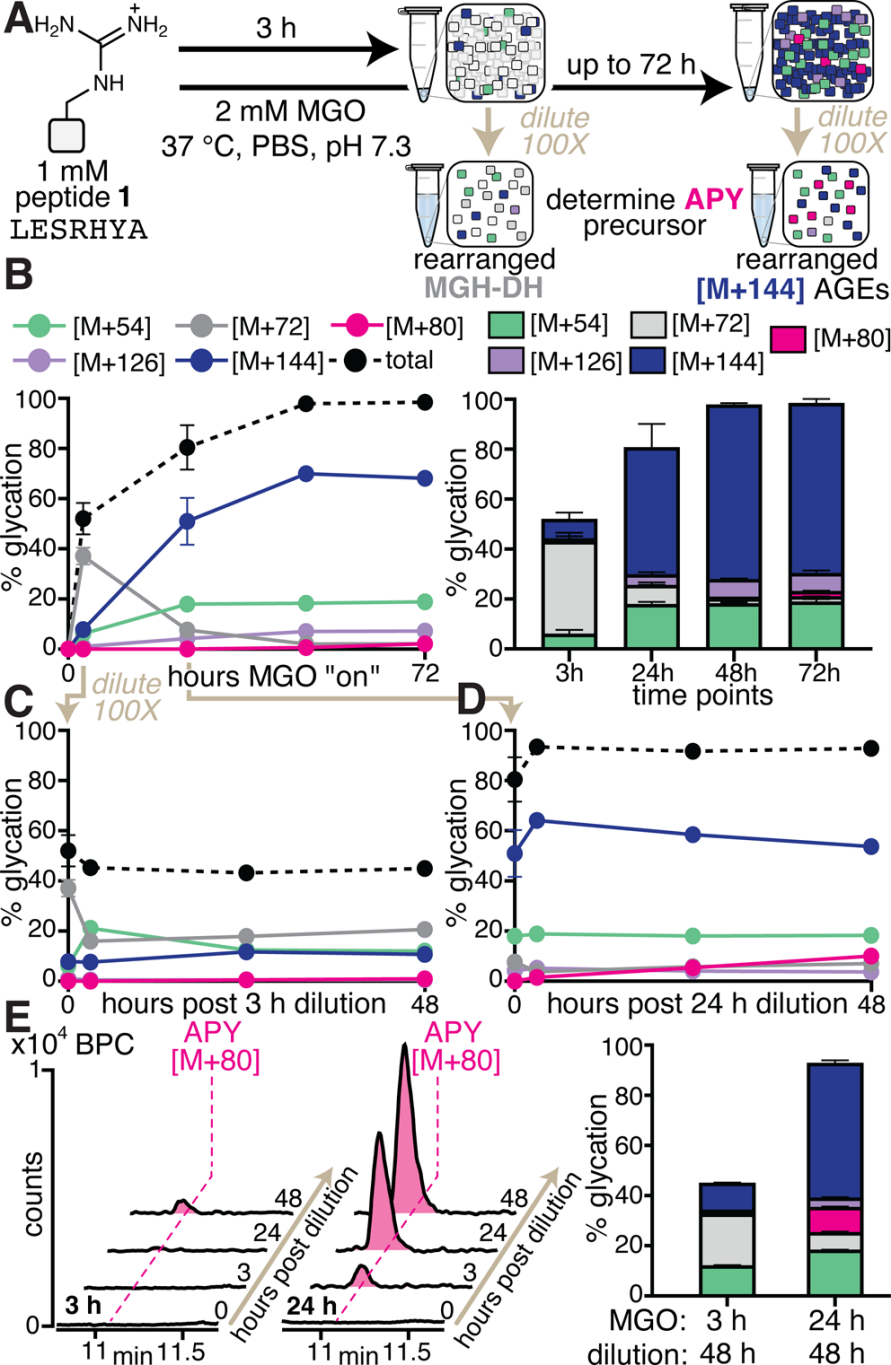
**[M + 144] AGEs are a Precursor to APY.** (A) General scheme showing the experimental setup used to determine the APY precursor. (B) Peptide **1** (1 mM) was treated with 2 mM MGO and incubated for up to 72 h (labeled as hours MGO “on”), as shown in a time course (left) and stacked bar graph (right) showing AGE distributions. (C) After 3 h, (when MGH-DH became the predominant adduct) or (D) after 24 h (when [M + 144] species become the predominant AGEs), the reaction was diluted 100× in the same buffer and incubated for additional 48 h post dilution (“hours post dilution”). Since dilution markedly slows MGO addition, this protocol diminishes new AGE formation on already glycated peptides, enabling the clear observation of rearrangements between AGEs. (E) Representative base peak chromatograms (BPC) (left) and bar graph (right) for peptide **1** at several post dilution time points (*n* ≥ 3 for all reactions). Please note that not all error bars are visible, as many are smaller than the size of the symbol. A significant increase in APY [M + 80] (magenta) levels was observed at 48 h post dilution of the 24 h time point when compared to that of the 3 h time point. These results suggest that [M + 144] species (blue), and not MGH-DH (gray), is a precursor for APY.

Nonetheless, the major difference in AGE distribution at 3 and 24 h allowed us to test the hypothesis that [M + 144] AGEs (likely including THP) are a precursor to APY. To do so, we incubated peptide **1** with 2 mM MGO for either 3 or 24 h. After this initial incubation, we diluted the reaction mixture 100× in the same buffer and compared the resulting AGE distributions at several post dilution time points (Figure 2C–E). Since dilution markedly slows MGO addition, this protocol diminishes new AGE formation on already glycated peptides, thereby enabling the clear observation of rearrangements between AGEs. At 3 h, MGH-DH (37.1 ± 3.4%) was the predominant AGE, but at 24 h, multiple [M + 144] species (51.0 ± 9.4%) became the predominant AGEs. While dilution of the 3 h incubation did not yield any APY even at 48 h post dilution (Figure 2C,E), we observed a substantial increase in [M + 80] levels when the 24 h incubation was diluted, reaching 10.0 ± 0.3% APY after 48 h post dilution (Figure 2D,E). These data confirmed the hypothesis that [M + 144] AGEs are a precursor to APY. We note that we consistently observed up to four different [M + 144] species with distinct retention times (Figures 3 and S2). To our knowledge, THP is the only [M + 144] AGE that has been reported.^26^ While it is possible that THP diastereomers may have different retention times, we suspect that these discrete [M + 144] adducts could also represent other intermediate structures or other double addition adducts (see also Figure 4).

**Figure 3:**
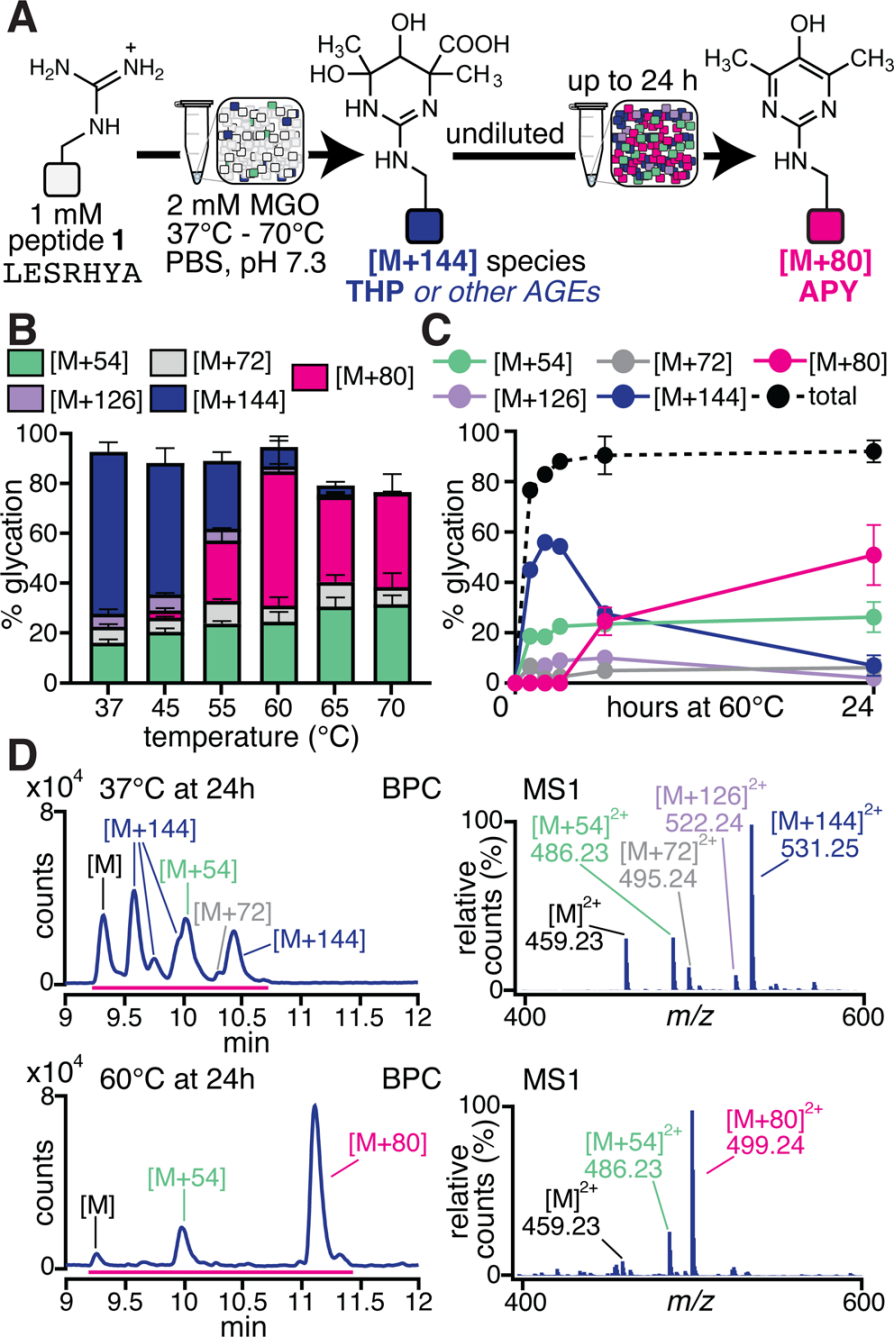
**APY is a stable AGE.** (A) Seeking to increase APY levels, we conducted a temperature scan by treating peptide **1** with 2 mM MGO over a range of temperatures from 37 to 60 °C. The reaction was incubated for 24 h undiluted, and AGE distributions were assessed via liquid chromatography–mass spectrometry (LC–MS) analysis. (B) Stacked bar graph showing AGE distributions for peptide **1** treated with MGO for 24 h at 37 °C, 45 °C, 55 °C, 60 °C, 65 °C, and 70 °C. At 55 °C, we began to observe a notable increase in APY [M + 80] (magenta) that was concomitant with reduced [M + 144] (blue) levels. APY was the predominant adduct in just 24 h at 60 °C. (C) Time course of peptide **1** glycation reactions at 60 °C, extended over several time points (1, 2, 3, 6, and 24 h) (*n* ≥ 3 for all reactions). Under these conditions, [M + 144] quickly became the predominant adduct in just 2 h, then decreased precipitously between 2 and 24 h. During this same time frame, a dramatic increase in APY levels was observed, confirming [M + 144] as a precursor to APY. Please note that not all error bars are visible, as many are smaller than the size of the symbol. (D) Representative BPCs and MS1 spectra for the glycation reaction of peptide **1** after 24 h incubation at 37 °C (top) and 60 °C (bottom). Together, these results provide conditions at which APY becomes the predominant AGE, suggesting that APY formation likely involves a relatively high energy activation barrier that can be overcome by the addition of heat.

**Figure 4:**
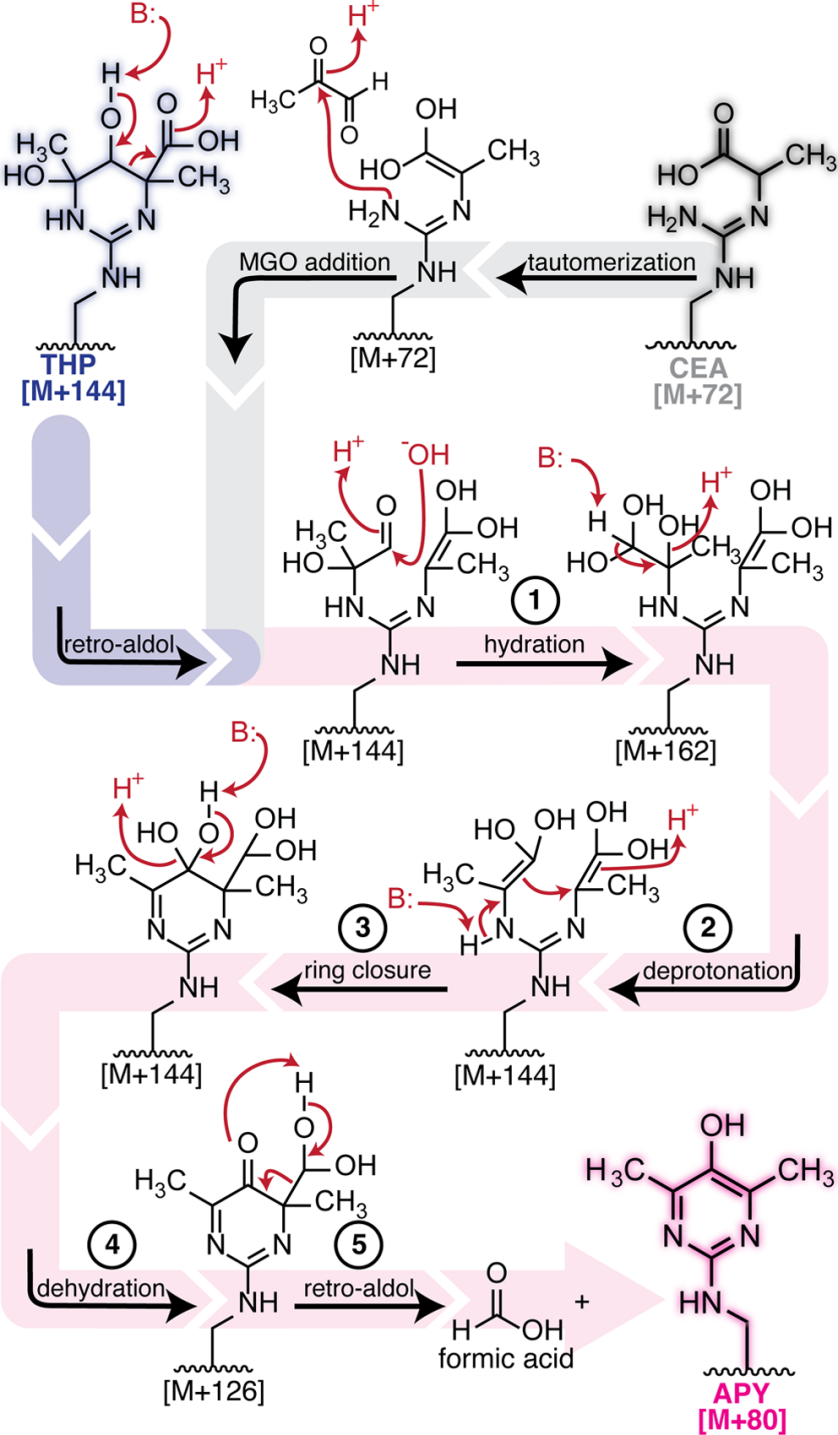
**Proposed mechanism for APY formation.** We propose that THP can rearrange into APY via a retro-aldol step (blue path), followed by hydration (1), then deprotonation (2) and ring closure (3), another dehydration step (4), and finally a retro-aldol step (5) that also releases formic acid as a byproduct (pink path). Alternatively, it is likely that other [M + 144] species could also rearrange into APY. Here we propose an alternate pathway, beginning from CEA (gray path), which involves tautomerization and addition of a second MGO equivalent to reach a common [M + 144] intermediate along the APY formation pathway.

### Enhancing APY Formation

Despite defining [M + 144] as an intermediate along the APY formation pathway, persistent low levels of APY remained a consistent technical challenge. Seeking to increase APY levels, we conducted a temperature scan in which we incubated peptide **1** with 2 mM MGO for 24 h, using temperatures ranging from 37 to 70 °C (Figure 3). We found that between 37 and 45 °C, there was hardly any change in the AGE distribution. Across the entire range of temperatures used, other AGEs, like MGH-1 and CEA, stayed fairly constant, with only slight increases at higher temperatures. However, at 55 °C, we began to observe a notable increase in APY that was concomitant with reduced [M + 144] levels. APY levels peaked at 60 °C (53.1 ± 12.1%), becoming the predominant AGE in just 24 h (Figure 3A,B). Due to high levels of APY observed at 60 °C, we were able to confirm its identity using its characteristic fluorescence wavelengths (ex = 320 nm, em = 385 nm) (Figure S3).^2,20,25,27^

To further investigate the relationship between APY and [M + 144], we extended the reaction at 60 °C across multiple time points. At 60 °C, the reaction quickly reached 88.1 ± 1.9% total glycation in only 3 h. From this, we conclude that most of the free MGO is quickly consumed, producing [M + 144] as the major early AGEs (54.3 ± 1.8% of total glycation) (Figure 3C). APY levels began to increase after 2 h of incubation, with a precipitous increase (from 0.0% to 24.5 ± 5.5%) between 3 and 6 h. During this same time frame, a concomitant, proportional decrease in [M + 144] levels was observed, providing unambiguous assignment of [M + 144] as a precursor to APY. Notably, all of the [M + 144] species we observed appear to mature into APY, since each one decreased at the same rate as APY levels increased (Figure S2). For this reason, we report all [M + 144] species totaled together, represented as a combined [M + 144].

Additionally, comparison of the AGE distributions at 37 and 60 °C revealed that APY is a stable AGE. After heating at 60 °C for 24 h, only MGH-1 and APY remained in appreciable quantities, while other AGEs, like CEA and [M + 144] species including THP, disappeared, presumably due to rearrangements into MGH-1 and APY (Figure 3D). We have also shown that while elevated temperatures increase APY levels at earlier time points, APY formation is still dependent on the sequence identity (Figure S4). Thanks to high levels of APY observed, we also conducted stability studies for purified APY on peptide **1** (peptide **1** ^**APY**^) and showed that APY on a short peptide is stable, exhibiting minimal decay for up to a week at physiological conditions (Figure S5). Importantly, these findings not only identify conditions at which APY can become a major AGE, but they also suggest that the rearrangement of [M + 144] into APY likely involves a high energy activation barrier that can be overcome by the addition of heat.

### A Proposed Mechanism for APY Formation

Next, we sought to understand possible mechanisms through which this rearrangement could take place. The most cited APY formation mechanism involves the generation of a reductone from two MGO, which then reacts with Arg to form APY (Figure S6).^2^ In this model, preincubation of MGO alone would be expected to generate the reductone, consequently leading to faster APY formation once added to peptide. We found that preincubation, even at strongly acidic pH and elevated temperatures (60 °C), did not promote any further APY formation upon addition to peptide **1** (Figures S7 and S8), and we did not observe any evidence of reductone formation by NMR (Figure S9). These results are therefore inconsistent with reductone formation as a plausible model for APY formation in physiological conditions. Another proposed mechanism suggests that MGH-3 rearranges to form APY, presumably through oxidative decarboxylation.^24^ However, our previous work shows that peptide **1** preferentially forms the MGH-1 isomer as confirmed by NMR, which we also confirmed in this study by matching the retention time (Table S1). As peptide **1** still forms APY, this suggests that MGH-3 is not a prerequisite for APY formation.^4^

Previous proposals have hypothesized that THP rearranges to APY through a pathway involving oxidation, followed by decarboxylation and then dehydration (Figure S6).^24,25^ To evaluate the feasibility of an oxidative decarboxylation from [M + 144], we first sought to understand whether APY could form under O_2_-free conditions. We scrupulously degassed all reaction components and used a glovebox to assemble and seal glycation reactions inside an N_2_ sparged (O_2_ < 4 ppm) scintillation vial that was further sealed prior to incubation. The resulting APY levels observed were identical in the presence or absence of oxygen (Figure S10), suggesting that a formal oxidation step may not be required. However, our studies were performed using commercial sources of MGO, which may contain impurities. To see if any potential impurities were contributing to APY formation, we synthesized MGO using an established Riley oxidation protocol (Figure S11).^28^ We found that there was no difference in APY formation between a freshly synthesized stock of MGO and commercial MGO. We also evaluated the role of buffer and salt concentrations, and found negligible differences (Figure S12).

As we could not attribute the source of oxidation required in the previously proposed mechanism, we considered other potential mechanisms in which THP—or other [M + 144] species—could rearrange to form APY. Beginning from THP, we identified a possible pathway (Figure 4) in which initial deprotonation of the hydroxyl group at C5 promotes a retro-aldol ring opening (Figure 4, blue path). Next, hydration of the aldehyde followed by deprotonation generates a geminal enediol at C5, followed by subsequent ring closure and another dehydration and retro-aldol step to generate the pyrimidine ring (Figure 4, pink path). This proposed mechanism generates formic acid as a byproduct. Supporting this proposal, we used an enzyme coupled assay to detect formic acid in reactions of peptide **1** with 2 mM MGO after 24 h (Figure S13). Our results indicate that formic acid is a byproduct of APY formation, as more formic acid was detected as APY formed on peptide **1** under our standard glycation conditions. However, we found that formic acid generation is not necessarily exclusive to APY, since we also detected formic acid when MGO was incubated with another sequence, (Ac-LDDREDA), that exhibited no detectable APY formation.

Our proposed mechanism involves an intermediate that includes a tautomer of CEA on one of the Arg Nω, allowing us to hypothesize that CEA could also be a precursor to APY. To evaluate this idea, we purified CEA and MGH-1 modified peptide **1** (peptides **1** ^**CEA**^ and **1** ^**MGH–1**^, respectively). At pH 7.3, without any MGO added, we observed only modest conversion from **1** ^**MGH–1**^ to **1** ^**CEA**^ with little degradation to peptide **1**, consistent with our previous results.^4^ Since peptide **1** does not form MGH-3 under our standard glycation reaction conditions, we do not rule out the possibility that MGH-3 can rearrange to form APY. However, upon treatment with additional equivalents of MGO, we found that peptide **1** ^**CEA**^ produced APY (Figure S14). This suggests that another source of the [M + 144] precursor to APY could be due to an additional MGO modification of CEA. This alternate pathway (Figure 4, gray path) shares a common intermediate with the one that formally begins with THP.

### APY Formation Requires a General Base

To further evaluate the feasibility of our proposed mechanism, we predicted that a general base would be necessary for the initial deprotonation step that promotes ring opening, as well as for subsequent ring closure and dehydration (Figure 4). Using a pH scan, we found that APY levels increased between pH 7.3–11 (Figure 5A,B). There was a particularly large jump in APY levels between pH 10 (3.3 ± 1.4%) and pH 11 (20.3 ± 1.1%), prompting us to conduct a narrower pH scan at 0.2 increments between pH 10 and 11. In this range, we found that APY levels increased steadily, peaking at pH 11 (20.3 ± 1.1% of total glycation) (Figures 5C and S15). There was a sharp transition between pH 10.2 and 10.4, suggesting a strong correlation with the protonation state of the nearby Tyr residue (p*K*_a_ 10.3).^29–31^ As a control, we also synthesized a variant of peptide **1** that replaced Tyr with Phe (peptide **1** ^**Phe**^). Compared to peptide **1**, peptide **1** ^**Phe**^ produced substantially less glycation overall and less APY formation (Figure S16). These results suggest that, for peptide **1**, Tyr plays an active role in the APY formation mechanism.

**Figure 5:**
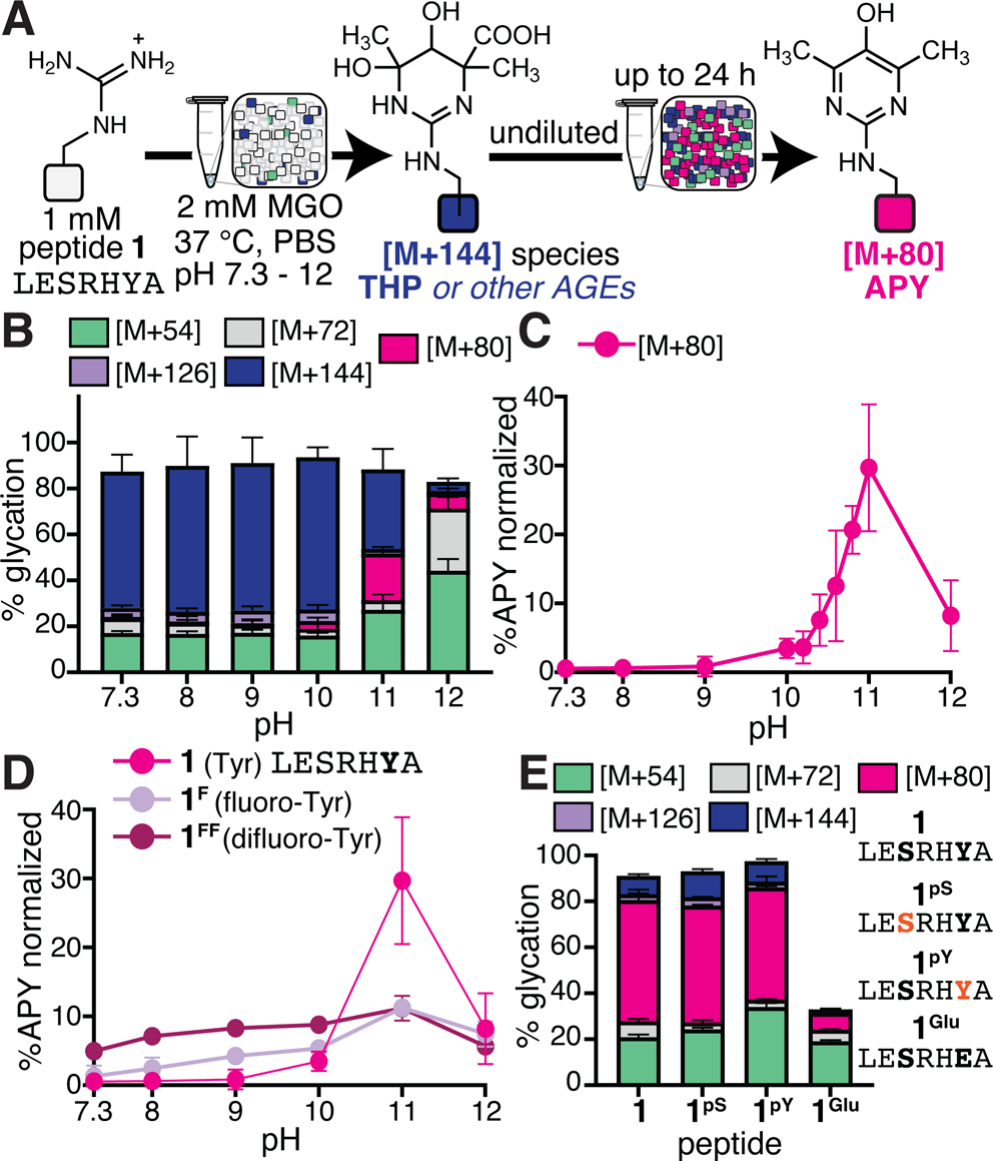
**A nearby general base facilitates the rearrangement of [M + 144] into APY.** (A) General scheme depicting a pH scan used to evaluate the features that promote APY formation. Peptide **1** (1 mM) was treated with 2 mM MGO using pH-adjusted phosphate buffered saline (PBS) in a pH range of 7.3–12. The reaction was incubated for 24 h undiluted at 37 °C. (B) Stacked bar graphs showing AGE distributions, as assessed by liquid chromatography–mass spectrometry (LC–MS) analysis. (C) Between pH 10 and 11, we observed a marked jump in APY [M + 80] (magenta) levels, as shown by proportional APY levels that were normalized to the total amount of glycation observed for MGO-treated peptide **1** across a narrower pH range between pH 10 and 11 at 0.2 increments. (D) To further test the hypothesis that Tyr phenoxide plays an active role in APY formation, we conducted glycation reactions on variants of peptide **1**, including peptide **1** ^**F**^ (fluoro-Tyr in lieu of Tyr) and peptide **1** ^**FF**^ (difluoro-Tyr in lieu of Tyr). Here, proportional APY levels were normalized to the total amount of glycation to account for differences in total glycation. At pH 7.3 and 37 °C, % APY was the greatest for peptide **1** ^**FF**^ (p*K*_a_ 7.2), followed by peptide **1** ^**F**^ (p*K*_a_ 8.4), and then peptide **1** (p*K*_a_ 10.3), consistent with our hypothesis. (E) Stacked bar graphs showing AGE distributions for glycation reactions with peptide **1**, peptide **1** ^**pS**^ (pSer in place of Ser, p*K*_a2_ 5.6), peptide **1** ^**pY**^ (pTyr in place of Tyr, p*K*_a2_ 5.9), and peptide **1** ^**Glu**^ (Glu in place of Tyr) at pH 7.3 and 60 °C (24 h incubation) (*n* ≥ 3 for all reactions). Despite the introduction of up to two negative charges which has been reported to hamper glycation, absolute APY levels and total glycation levels remained virtually identical for the phosphorylated variants. Together, these results demonstrate the importance of a nearby general base to facilitate APY formation and suggest that nearby phosphorylated residues can also aid glycation.

To test the hypothesis that Tyr phenoxide facilitates APY formation, we synthesized variants of peptide **1** that incorporated noncanonical Tyr derivatives with lowered phenolic p*K*_a_’s. Specifically, 3-fluorotyrosine (peptide **1** ^**F**^) and 3,5-difluorotyrosine (peptide **1** ^**FF**^) were chosen because of their p*K*_a_ values (8.4 and 7.2, respectively) (Figure 5D).^32^ These peptides were reacted with 2 mM MGO across a range of pH between 7.3 and 12 (Figures 5D & S15), revealing maximal APY levels at pH 11 for all three peptides. However, when normalized to account for differences in total glycation levels, we found that proportional APY levels at pH 7.3 were greatest for peptide **1** ^**FF**^ (5.0 ± 0.3%), followed by **1** ^**F**^ (1.3 ± 1.5%), and then peptide **1** (0.6 ± 0.4%) (Figure S15). These results demonstrate the importance of the nearby Tyr as a facilitator for [M + 144] rearrangement into APY, matching our hypothesis that Tyr can act as a general base that facilitates APY formation.

Following the same logic, we next considered if phosphorylated Tyr—or perhaps even Ser or Thr—might also act as a general base promoting APY formation. Phosphorylated Tyr has experimental p*K*_a2_ values ranging between 4.0 and 6.1.^33^ Thus, we synthesized peptide **1** ^**pY**^ that contains pTyr in place of Tyr. After glycation with 2 mM MGO at pH 7.3 at 60 °C for 24 h, there were hardly any differences in total glycation and APY levels between peptide **1** (52.8 ± 4.5% [M + 80] at 24 h) and **1** ^**pY**^ (48.8 ± 0.6% [M + 80] at 24 h) (Figures 5E and S17). We performed a similar set of experiments using a peptide **1** variant with phosphorylated Ser (peptide **1** ^**pS**^), and found that these also produced comparable levels of glycation (50.9 ± 0.3%) after 24 h, despite the addition of up to two negative charges (Figures 5E and S17). By contrast, a variant of peptide **1** that replaced Tyr with Glu (peptide **1** ^**Glu**^) exhibited substantially less overall glycation and APY formation than peptide **1** (Figures 5E and S16).

To further examine this behavior, we evaluated the glycation of peptides **1**, **1** ^**pY**^, **1** ^**pS**^, and a doubly phosphorylated version (peptide **1** ^**pSpY**^) at 37 °C, over time (Figure S18). Supporting our findings at 60 °C, these studies showed APY levels to be similar across all times for peptides **1**, **1** ^**pY**^, and **1** ^**pS**^. However, MGH-1 levels were noticeably increased—especially at earlier times—for the phosphorylated substrates. Additionally, introduction of up to two negative charges on peptide **1** ^**pY**^ and **1** ^**pS**^ did not affect overall glycation but led to increased MGH-1 formation. However, at both lower and elevated temperatures, the introduction of four negative charges on peptide **1** ^**pSpY**^ decreased overall glycation (Figures S17 and S18). Our previous work has unambiguously shown that multiple negative charges surrounding glycation sites greatly impair AGE formation (Figure 5E).^4,5,34^ Therefore, this observation suggested to us that nearby phosphates may exert a positive influence on AGE formation, thereby counteracting any attenuation from their negative charges. Accordingly, these results imply that nearby phosphorylation may be helpful for glycation, and based on our proposed mechanism, could be particularly important for APY formation in a cellular context.

### Uncovering a Connection between Cellular Glycation and Phosphorylation

Guided by these chemical mechanistic insights, we performed a quantitative proteomics experiment using HEK-293T cells treated with or without MGO. Our mechanistic studies have shown that APY can accumulate at longer times or using higher MGO concentrations. Therefore, we opted to treat cells with 2 mM MGO for 24 h to ensure that we could induce APY formation. We confirmed that these treatment conditions did not significantly decrease cell viability, as more than 95% of cells remained viable after treatment (Figure S19). Several reagents, such as dithiothreitol (DTT) and iodoacetamide, used in proteomic experiments were tested for their effects on AGE distribution and APY formation and found to have no interference (Figure S19). Additionally, as there are no highly specific antibodies available to enrich APY (Figure S20), we chose to label an unenriched cell lysate with isobaric tandem mass tags, allowing us to evaluate the distribution of multiple AGEs using quantitative bottom-up proteomics, including the MGH isomers ([M + 54]), APY ([M + 80]), THP or other [M + 144] AGEs, and an [M + 72] adduct that could correspond to MGH-DH, CEA, or CEL. Using this approach, we identified 7210 total proteins and 56490 peptides across all conditions tested, and found 478 unique peptides that were AGE-modified upon treatment with 2 mM MGO. This data set shows significant similarity to other MGO-treated proteomic data sets, with AGE modifications enriched on proteins involved in processes such as protein folding and spliceosome function, along with several modified histone and heat shock proteins.^9,10,35,36^

Upon meticulous inspection of our data set, we manually removed any hits from the glycated set that had a Δ mass (theoretical – observed mass) > 20 ppm and/or were assigned with a modified Arg at their C-terminus, as we and others have previously shown that glycated Arg cannot be cleaved by trypsin.^5,37^ After data processing, this decreased the total list of AGE-modified peptides to 204 plausible AGE-modified hits (Supporting Information Excel File 1). Without any enrichment steps during sample preparation that could bias the AGE distribution, we found that APY and [M + 144] species were the predominant AGEs in the MGO-treated condition (57 unique peptides, 28%, and 53 unique peptides, 26%, respectively), followed by the MGH isomers and CEA/MGH-DH (47 unique peptides, 23% for each) (Figure 6B). Although our dynamic modification for [M + 72] (mass change 72.021) was set for both Arg and Lys, we only observed modification on Arg (MGH-DH and/or CEA, not CEL), likely due to the static TMT modification that was set for Lys in our processing workflow. Though glycation is largely driven by chemical microenvironments that include 3D protein structure and is therefore unlikely to have a strong consensus sequence,^34^ we were also able to obtain consensus features for each AGE (Figure S21).

**Figure 6:**
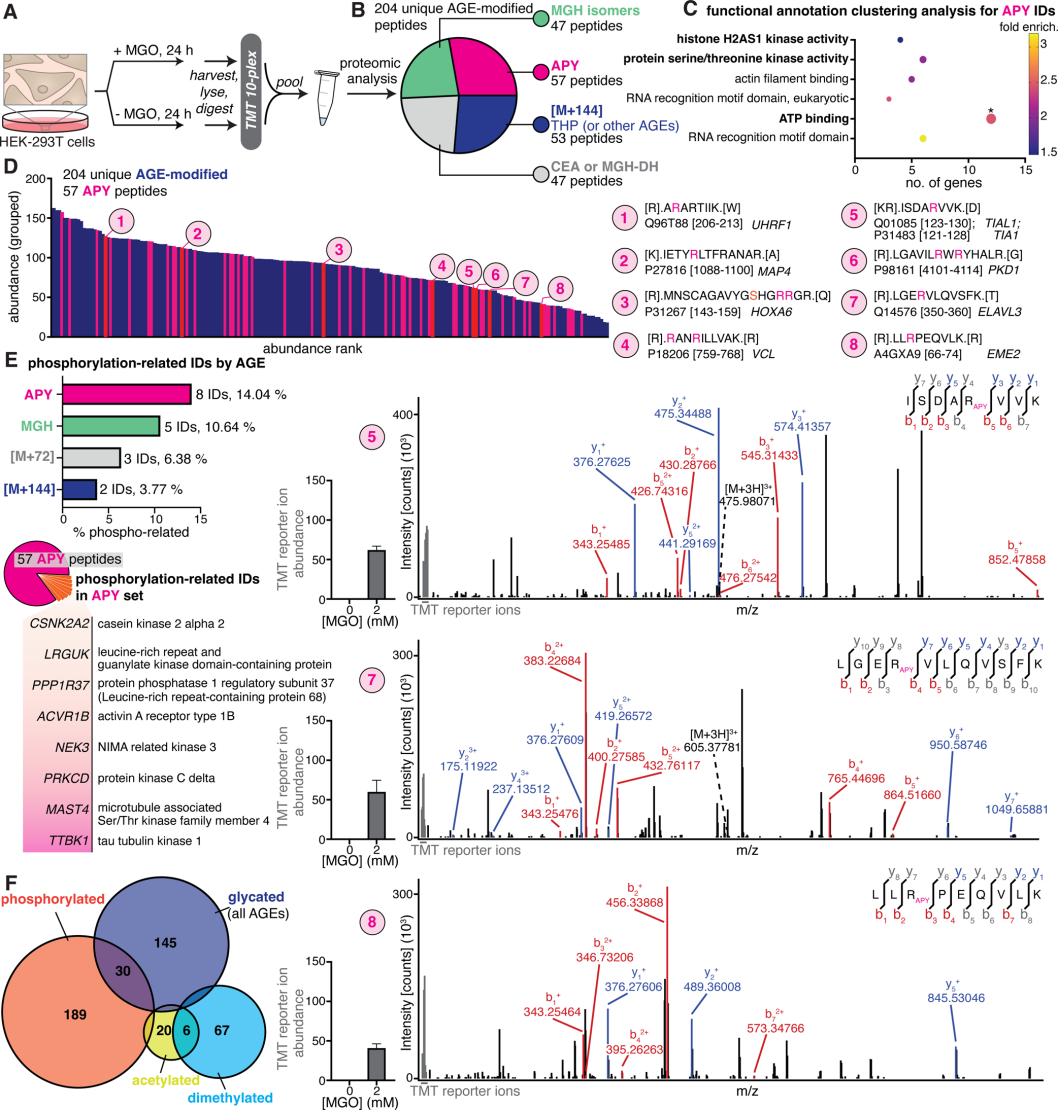
**Proteomic analysis on MGO-treated cells reveal a connection between glycation and cellular phosphorylation events.** Given that our proposed mechanism for APY formation was supported by in vitro peptide data, we set out to investigate APY formation in a cellular context, along with other common MGO-modified AGEs. (A) General scheme describing our proteomic workflow on HEK-293T cells treated with or without 2 mM MGO for 24 h. Whole cell lysates were trypsin-digested, labeled with TMT isobaric tags, and analyzed in a quantitative bottom-up workflow. The untreated sample was used as a frame of reference to identify any AGE increase in the treated condition. (B) Pie chart showing a set of 204 unique peptides with AGE-modified Arg in the MGO-treated condition, consisting of 57 APY-, 53 THP- (or other [M + 144] AGEs), 47 MGH-, and 47 CEA/MGH-DH-containing peptides. (C) DAVID functional annotation clustering analysis was performed for protein IDs associated with the APY set using a 0.05 enrichment threshold. The top term for each cluster is represented, and values for −log­(*p*-adjust) were calculated using reported Benjamini values from DAVID. Multiple phosphorylation-related clusters and terms (bolded text) were observed that are unique to the APY set. The top term for ATP-binding (*) from this analysis showed a statistically significant −log­(*p* adjust) value of 1.70. (D) The abundance (grouped) for APY-containing peptides (magenta) was ranked and displayed atop the abundance of other AGE-containing peptides (blue). Eight statistically significant increased APY-containing peptides (red) are numbered 1–8. Representative b/y ion spectra and TMT reporter ion abundance quantitation for three of the high confidence APY hits are shown. (E) Comparison of the percent of phosphorylation-related IDs by AGE type (top) and a list of the eight phosphorylation-related IDs observed in the APY set. (F) Venn diagram showing overlapping IDs between AGEs or other common Arg modifications (dimethylation and acetylation) and phosphorylation. Compared to dimethylated and acetylated IDs, glycated IDs exhibited substantial overlap (30 IDs) with phosphorylated IDs, further suggesting a connection between AGE formation and phosphorylation events.

Next, we performed gene ontology (GO) analysis for the APY-modified set of proteins using DAVID. In both the biological process (BP) and molecular function (MF) annotations, APY-containing proteins showed multiple terms related to phosphorylation, including protein phosphorylation (BP) and multiple kinase activities (MF) (Figure S22). We also found that none of the other AGE sets showed terms related to protein phosphorylation (BP) or kinase activity (MF) (Figures S22–S25). However, due to the relatively small number of hits, the depth of our proteomic analysis was not sufficient to gain meaningful statistical information. Despite the small size of our list of hits, we performed functional annotation clustering analysis, which groups similar and/or related annotations into a focused snapshot of biologically relevant clusters.^38^ From this analysis, the APY set was the only one to show clustering, producing six clusters with enrichment scores ≥1.3. Out of these six clusters, three were phosphorylation-related including ATP-binding (enrichment score 2.43), protein serine/threonine kinase activity (enrichment score 2.02), and histone kinase activity (enrichment score 1.48) (Supporting Information Excel File 2). Indeed, the top term for ATP-binding from this analysis showed a statistically significant −log­(*p* adjust) value of 1.70 (Figure 6C). We also used an alternative database (ToppGene) and manual annotation and ID mapping (UniProtKB) (Supporting Information Excel File 3). Similarly, only the APY set showed multiple phosphorylation-related clusters and terms, whereas other AGEs did not.

Next, we further investigated the set of 57 modified APY-containing peptides, which were observed across the entire range of AGEs when ranked by abundance (Figure 6D). Among this list of genes, eight (14.04%) were annotated with phosphorylation-related GO terms, such as ATP binding or kinase activity (Figure 6E). Compared to other AGEs such as the MGH isomers, CEA or CEL, and [M + 144] species, the APY set exhibited the greatest proportion of hits that were annotated with phosphorylation-terms. Taken together, to us these analyses suggested a possible connection between APY formation and phosphorylation in a cellular context.

To further evaluate the possibility of a connection between protein phosphorylation and APY formation, we focused in particular on the eight highest confidence APY-modified peptides that exhibited statistically significant increases in abundance upon MGO treatment across all three biological replicates (Figures 6D and S26–S30). Data imputation and statistical analysis were performed according to published protocols.^39^ Among these peptides, one was also identified with a phosphorylated residue nearby the APY-modified site, corresponding to HOXA6 (hit 3) (R156, R157 with nearby pS153).

Furthermore, by cross-referencing the protein sequences, reported phosphorylated sites, and the influence of phosphorylation on protein functions, we were able to draw several correlations between reported studies and our data set. Of the remaining seven high confidence APY hits, six were previously reported to be phosphorylated, and some are known to have their functions regulated by phosphorylation.^40^ For example, phosphorylation of MAP4 (hit 2) has been reported to affect microtubule properties and cell cycle progression, while TIAL1 (hit 5) has also been found to be phosphorylated following DNA damage, and phosphorylation of PKD1 (hit 6) triggers its membrane dissociation and subsequent entry into the nucleus.^41–43^ Additionally, phosphorylation of UHRF1 maintains protein stability, subcellular localization, and epigenetic regulation (hit 1).^44^ Careful inspection of our data revealed that UHRF1 was APY-modified at R207, which is close to T210, a known phosphorylated site previously reported to be essential in maintaining UHRF1 stability, though phosphorylated T210 was not observed in our data set.^45^

Similarly, S358, close in sequence to APY-modified R353, on ELAVL3 (hit 7) has also been shown to be phosphorylated.^40^ We also found two APY modifications on the C-terminal domain of PKD-1 (R4107, R4109), which contains 4 Tyr residues (Y4110, Y4118, Y4127, Y4237) and 2 putative Ser sites (S4169, S4252) that are susceptible to phosphorylation. Multiple in vitro studies have revealed Y4237 and S4252 to be targets of phosphorylation by pp60c-src and PKA, respectively, and shown endogenous PKD-1 to be phosphorylated at tyrosine.^46–48^ Together, our data show several APY modifications on Arg residues close to known phosphorylation sites, consistent with the potential for crosstalk between cellular APY and phosphorylation events.

However, as APY (mass change 80.026) is quite close in mass to phosphorylation (mass change 79.966), we also queried our data set for phosphorylation and found that the list of proteins generated had some overlap with the set of APY-modified targets, but the overall list of IDs was quite different, confirming that the relatively similar mass changes did not produce artifactual hits. We also found that the phosphorylated set (220 proteins) showed substantial overlap (30 proteins) with the AGE-modified set of proteins (180 proteins). To evaluate the possibility that crosstalk between nearby phosphorylation could promote AGE formation (especially for APY and MGHs), we performed a parallel analysis for other common Arg PTMs, including dimethylation and acetylation (Figures 6F, S31 and S32). We found 99 unique peptides with dimethylated Arg and 30 unique peptides with acetylated Arg. Importantly, the phosphorylated set of proteins did not substantially overlap with those that were observed to be dimethylated (9 IDs) or acetylated (2 IDs) (Figure 6F).

### Confirming that Phosphorylation can Potentiate Glycation

Overall, our proteomic data set supported our in vitro experiments, suggesting that APY formation might be promoted by phosphorylated residues that act as general bases to facilitate rearrangement into APY (Figure 4). To validate our findings, we synthesized three peptides matching APY-modified sequences in our data set. These include a sequence from MAP4 (hit 2; peptide **V1**: Ac-ETYRLTF), and one from ELAVL3 (hit 7, peptide **V2**: Ac-LGERVLQ). We also synthesized a peptide from HNRNPA3, which was found in higher abundance than either hit sequence, though it did not meet our threshold for being considered “high confidence” (peptide **V3**: Ac-YNLRDYF). Upon treatment with 2 mM MGO at 60 °C for 24 h, we confirmed that all three peptides formed APY (Figure 7A,B). Expectedly, peptide **V2**, without a nearby Tyr, exhibited the least APY formation out of the three peptides, though we note that it produced more APY than control sequences such as peptide **1** ^**Glu**^ and peptide **1** ^**Phe**^. Matching our expectations, we found that the peptide with two Tyr residues (peptide **V3**) exhibited the most APY formation. We also found that the overall extent of glycation and the amount of APY formed for peptide **V1** and **V3** were quite similar to that observed for peptide **1**. However, as our treatment condition at 60 °C for 24 h does not reflect the initial rate of APY formation, we also monitored the AGE distributions at 37 °C at 24 and 48 h (Figures 7C and S33). Under these conditions, peptide **V1** exhibited the most APY formation. This behavior was visible at earlier time points, and was particularly apparent after 48 h of treatment. Importantly, these data validate that peptides matching the APY-modified sequences identified in our proteomic analysis are indeed capable of forming APY in vitro.

**Figure 7:**
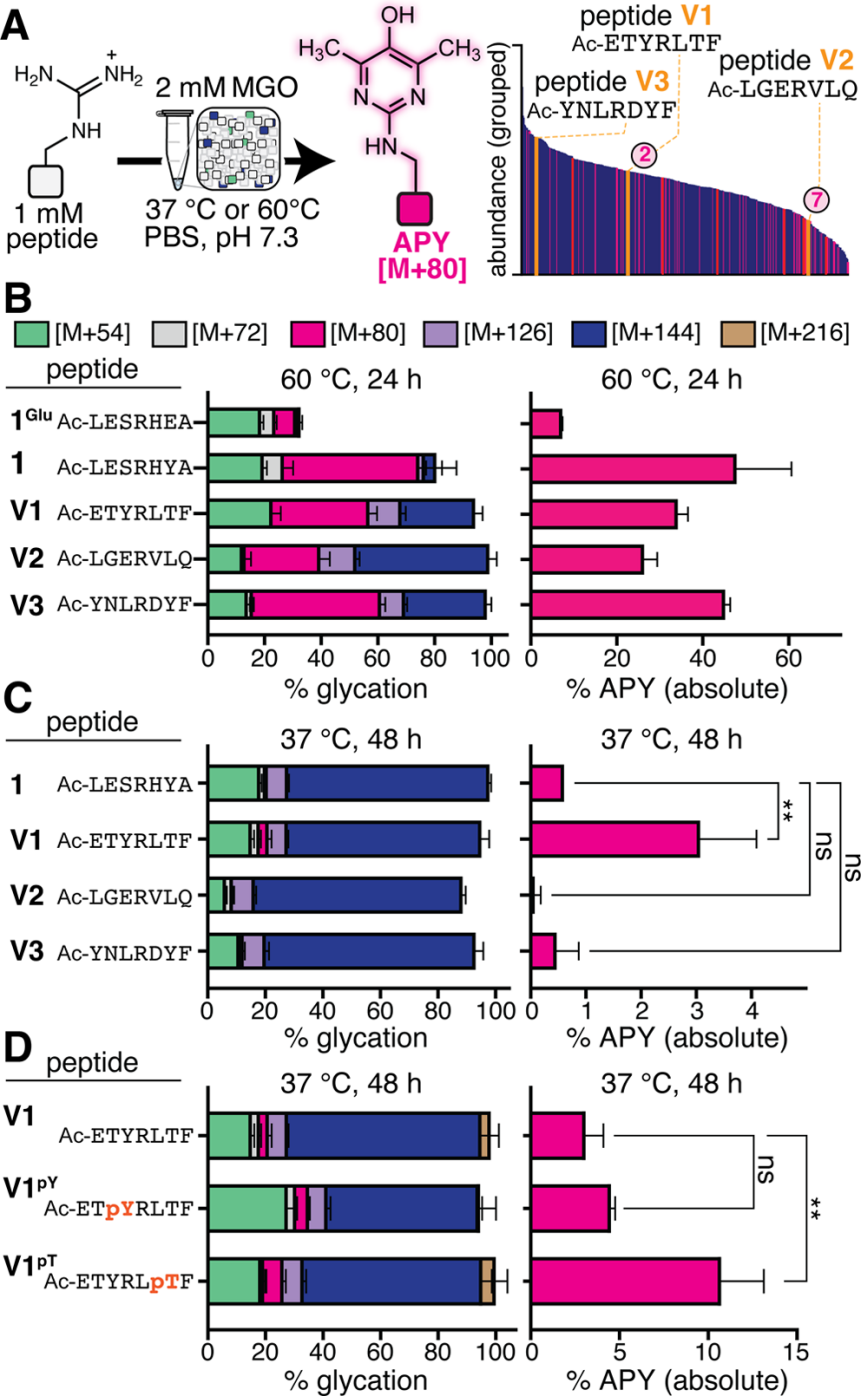
**Confirming that phosphorylation can potentiate APY formation using validated hit sequences in vitro.** (A) To validate our proteomics analysis, we synthesized peptides matching APY-modified hit sequences in our data set: Ac-ETYRLTF (hit 2; peptide **V1**), Ac-LGERVLQ (hit 7; peptide **V2**), and Ac-YNLRDYF (peptide **V3**), a high-abundance sequence. These peptides were evaluated after treatment with MGO. (B) Distributions of AGEs at 60 °C after 24 h of MGO treatment (left) for peptides **1**, **V1**, **V2**, **V3**, and **1** ^**Glu**^. Absolute APY levels (%) observed under the same conditions (right) (*n* = 3). All three peptides identified from proteomic analysis were confirmed to form APY. (C) Distributions of AGEs at 37 °C after 48 h of MGO treatment (left) for peptides **1**, **V1**, **V2**, and **V3**. Absolute APY levels (%) observed under the same conditions (right) (*n* = 3) show that peptide **V1** exhibits the most APY formation. Ordinary one-way ANOVA was used to determine if each variant yielded statistically significant differences in % absolute APY compared to peptide **1**. *p* < 0.01­(**). (D) Distributions of AGEs at 37 °C after 48 h of MGO treatment (left) for peptides **V1**, **V1** ^**pY**^, and **V1** ^**pT**^. Absolute APY levels (%) observed under the same conditions (right) (*n* = 3). All three peptides exhibited similar levels of overall glycation. Peptide **V1** ^**pT**^ was the fastest to form APY, followed by peptide **V1** ^**pY**^, and then peptide **V1**. Ordinary one-way ANOVA was used to determine if each variant yielded statistically significant differences in APY levels compared to peptide **V1** (*n* = 3). *p* < 0.01­(**). Absolute APY levels in peptide **V1** ^**pT**^ were 3-fold higher than those in peptide **V1**, and both phosphorylated variants exhibited an increase in MGH-1 formation, indicating that phosphorylation has a direct influence on glycation outcomes.

As peptide **1** was identified from a prior study focused on MGH-1, we found that introduction of nearby phosphates appeared to have an impact on MGH-1, rather than APY, formation. To determine if this was also the case for hit peptides, we synthesized two variants of peptide **V1** that incorporated either pTyr or pThr (Ac-ETpYRLTF, peptide **V1** ^**pY**^, and ETYRLpTF, peptide **V1** ^**pT**^) (Figures 7D and S33). At 60 °C for 24 h, all three peptides exhibited similar levels of overall glycation and APY formation, despite the introduction of extra negative charges. Compared to peptide **V1** ^**pT**^ and nonphosphorylated peptide **V1**, peptide **V1** ^**pY**^ showed a significant increase in MGH-1 formation. However, at 37 °C, peptide **V1** ^**pT**^ was the fastest to form APY, followed by peptide **V1** ^**pY**^, and then peptide **V1** (Figure 7D). Absolute APY levels in peptide **V1** ^**pT**^ were 3-fold higher than those in peptide **V1**. Interestingly, similar to the trends observed for peptide **1** and its phosphorylated variants, peptide **V1** ^**pT**^ and **V1** ^**pY**^ also exhibited an increase in MGH-1 formation, with absolute MGH-1 levels in peptide **V1** ^**pY**^ observed to be approximately 2-fold higher than those in peptide **V1** (Figures 7D and S33), indicating that phosphorylation can also bias the formation of certain AGEs. Taken together, these data support a model in which crosstalk with protein phosphorylation could have an important, but previously underappreciated role in regulating cellular glycation events.

## Discussion

Herein we have used a peptide model system to determine a chemical mechanism for APY formation. Although appreciated for its unique fluorescence properties,^2,24,49^ APY has been difficult to study as it was not previously known how to produce it in experimentally convenient quantities from the reaction of Arg and MGO.^17,18,20^ Our results indicate that APY formation depends surprisingly little on initial MGO concentrations, and instead greatly depends on other factors, including sequence identity, reaction pH, and temperature, each of which can help to overcome a relatively high energy rate-determining step. Through these studies, we discover that APY is quite stable and identify in vitro conditions under which APY becomes the predominant AGE, which to our knowledge, has not been previously reported. These findings alone provide an important technical advance that will greatly facilitate future studies on APY.

Additionally, our proposed mechanism relies on fundamental concepts of organic chemistry and provides concrete experimental evidence that reconciles several previous, conflicting mechanisms proposed for APY. While others have suggested that THP is a precursor to APY, our newly proposed mechanism circumvents the need for an unexplained, mysterious oxidation step by recognizing that THP possesses a β-hydroxycarbonyl, a common feature in retro-aldol substrates. Indeed, the retro-aldol reaction is one of the most important organic reactions in sugar fragmentations, including those in physiological systems.^50–52^ Several known retro-aldol enzymes use Tyr as a general base,^53–55^ which aligns with our results showing noncanonical Tyr variants with decreased phenolic p*K*_a_ to promote APY formation. However, we also identify a potential alternative pathway involving highly plausible [M + 144] intermediates other than THP. While we cannot definitively confirm all steps in our proposed mechanism, it is fully consistent with our experimental results ruling out other previously proposed alternatives. Additionally, we note that most steps are precedented in biological systems, including the retro-aldol reaction^50–52^ and tautomerization of hydrated aldehyde consistent with glyoxalase activity.^56–59^ Our mechanism also connects APY formation to the emancipation of formate, a major reactive species in one-carbon metabolism that is involved in numerous biological processes.^60–62^ While there has been some prior work connecting the metabolic processing of MGO to formate generation,^63–67^ our results suggest that cellular glycation events themselves, including but not limited to APY formation, may also contribute to the cellular formate pool.

Encouragingly, our data set shows that APY is likely to be a major AGE in cells. We suspect the prevalence of APY has seemingly been underestimated in previous studies, either due to the use of enrichment protocols that are poorly suited to capture APY,^68,69^ or due to a focus on AGEs that were previously established to be abundant in prior work.^70,71^ Our mechanistic studies therefore not only provide much needed insights into APY formation under physiologically relevant conditions, but also reveal critical aspects of the glycation landscape. In future studies, we plan to leverage this information to generate novel chemical tools that can be used to track and/or control APY formation in living cells, including the development of tools that harness APY’s unique autofluorescent properties for bioconjugation and to assist in the study of APY biology.

This study also reveals the potential for crosstalk between glycation and phosphorylation, showing the presence of nearby phosphorylated side chains to enhance, and potentially bias, particular AGEs to form. Our experiments examine the ability of phosphorylated residues (pSer, pTyr, and pThr) to promote formation of certain AGEs, particularly that of MGH-1 and APY, on nearby Arg despite introducing additional negative charges previously shown to hamper glycation.^4,5^ These peptide data are also supplemented with quantitative proteomics analysis on MGO-treated cells, revealing a possible correlation between phosphorylation and APY formation that was notably missing for other AGEs and Arg PTMs like acetylation and dimethylation. This correlation was further confirmed using in vitro glycation experiments on phosphorylated peptide substrates. While inorganic phosphate has long been known to enhance glycation,^72^ and MGO has been shown to stimulate phosphorylation,^73–76^ we have not been able to find any reports suggesting that nearby phosphorylated sites influence glycation. Therefore, our study is the first to show that glycation may be coincident with phosphorylation on the same substrates, and that the presence of these nearby phosphates may bias the resulting AGE distribution. Our future work will seek to fully elucidate both the chemical and biological significance of this phenomenon.

## Methods

### General Materials

All chemical reagents and solvents were of analytical grade, obtained from commercial suppliers, and used without further purification, unless otherwise noted. Methylglyoxal (MGO) (40% w/v in water) was purchased from MilliporeSigma (M0252). *N*-Fmoc-3-fluoro-l-tyrosine (1270290) was purchased from Acrotein Chem Bio Inc. *N*-Fmoc-3,5-difluoro-l-tyrosine (AS01393) was purchased from Acrotein Chem Bio Inc. Fmoc-Tyr­(PO­(OBzl)­OH)-OH (FY2580) was purchased from Advanced ChemTech Inc. Fmoc-Ser­(HPO3Bzl)-OH (101604) was purchased from ChemPep Inc. Aminoguanidine hydrochloride (396494), NAD (10127981001) and NADH (10128023001) standards were purchased from Sigma-Aldrich. Formic acid assay kit was purchased from Neogen Corporation (K-FORM). HEK-293T cells were purchased from ATCC. Water used in glycation experiments was distilled and deionized using an Arium Pro purification system (Sartorius). All statistical analysis was conducted using Prism GraphPad.

### Peptide Synthesis

Peptides were synthesized using standard Fmoc-based solid phase peptide synthesis on Trityl chloride resin (200 mesh, 1.6 mmol/g loading, Novabiochem) or Fmoc-Ala-Wang resin (100–200 mesh, 0.64 mmol/g loading, Creosalus Inc.), typically on a 100 μmol scale in a 3 mL polypropylene fritted syringe. For trityl chloride resin only, C-terminal amino acids (5 equiv, 500 μmol) were coupled overnight in a solution of 10 equiv (1 mmol) *N*,*N*-diisopropylethylamine (DIEA) in 3 mL dimethylformamide (DMF). The next day, the resin was washed and capped with a solution (3 mL) of DMF/MeOH/DIEA (17:2:1 by volume) for 1 h before further deprotection and coupling steps. C-terminal coupling and resin capping was not required when using Fmoc-Ala-Wang resin. Fmoc deprotection was accomplished using 20% piperidine in DMF (3 mL, 3 × 5 min), followed by washing steps with DMF (3 mL, 4 × 1 min). Amino acid couplings were completed by incubation of amino acid (5 equiv relative to resin loading) with *O*-(benzotriazole-1-yl)-*N*,*N*,*N*′,*N*′-tetramethyluronium hexafluorophosphate (HBTU, 5 equiv) and DIEA (10 equiv) in 1–2 mL of DMF for 45–90 min. To prepare for pTyr and pSer couplings, deprotection steps right before and after each coupling step were extended to 3 × 10 min, and the couplings were extended to 2–3 h. All peptides were N-terminally acetylated following deprotection of the last amino acid coupling via incubation with acetic anhydride (4 equiv) and DIEA (3 equiv) in 1–2 mL DMF for 2 h. Following the final acetylation step, peptides were washed with DMF (3 mL, 4 × 1 min), followed by dichloromethane (DCM) (3 mL, 2 × 1 min, then 2 × 15 min), and stored under vacuum desiccation until ready for side-chain deprotection and cleavage. The side chain protecting groups used were as follows: Glu (*t*Bu), His (Trt), Lys (Boc), pSer (Bzl), pTyr (Bzl), Trp (Boc), and Tyr (*t*Bu). Side-chain deprotection and peptide cleavage was completed by incubation with trifluoroacetic acid (TFA), triisopropylsilane (TIPS), and water (95:2.5:2.5 by volume) in 4 mL of acid solution per resin for 2 h. The resulting crude peptide was dried under constant air flow and then dissolved in 1–3 mL of a water/acetonitrile mixture based on solubility, prior to purification.

### Peptide Purification

All peptides were purified using a semipreparative Agilent 1260 Infinity LC system equipped with an Agilent ZORBAX SB-C18 column (9.4 × 250 mm, 5 μm particle size). The mobile phase contained water (A) and acetonitrile (B) with 0.1% TFA. Crude peptide solutions were eluted with a gradient of 5% B to 40% B over 20 min and a flow rate of 3.0 mL/min. Crude peptide solutions of pTyr- and pSer-containing peptides were eluted with a gradient of 5% B to 35% B over 23 min and a flow rate of 2.0 mL/min. Absorbance at 215 and 280 nm was used to observe desired peptide peaks, which were then eluted and collected using an automated fraction collector. APY-specific absorbance could be detected using its characteristic absorbance at 320 nm.^2,24,49^ Collected fractions were assessed for purity using matrix-assisted laser desorption/ionization time-of-flight (MALDI-TOF) mass spectrometry (Bruker) and/or an Agilent 6530 quadrupole time-of-fight (Q-TOF) mass spectrometer (Agilent). Pure fractions were combined and lyophilized. Peptide stock solutions were prepared at 20 mM concentrations in DMF. Peptide stock solutions of peptides containing multiple phosphorylated amino acid on the same sequence were prepared at 10 mM concentrations in 10 mM phosphate buffer (pH 10.0).

### MALDI Mass Spectrometry

0.5 μL of each peptide collected fraction were cocrystallized onto a ground steel plate with 0.9 μL of saturated solutions of α-cyano-4hydroxycinnamic acid in 50% acetonitrile, 50% water with 0.1% trifluoroacetic acid.

### Liquid Chromatography–Mass Spectrometry Analysis and Quantification

Reversed-phase liquid chromatography and mass spectrometry (LC–MS) was performed using an Agilent 1260 LC system coupled to an Agilent 6530 Accurate Mass Q-TOF. The mobile phase contained water (A) and acetonitrile (B) with 0.1% formic acid. Peptide glycation reactions were injected onto an AdvanceBio Peptide 2.7 μm column (2.1 × 150 mm, Agilent) with the following method with a flow rate of 0.4 mL/min: isocratic at 5% B between 0.00 and 1.75 min, gradient change from 5% B to 40% B between 1.75 and 16.00 min, gradient change from 40% B to 100% B between 16.00 and 20.00 min, isocratic column washing at 100% B between 20.00 and 23.00 min, and re-equilibration at 5% B between 23.01 and 30.00 min. Peptide data were quantified using peak volumes determined by Agilent MassHunter Qualitative Analysis and the MassHunter Molecular Feature Extractor based on cumulative MS ion counts for “volumes” observed for any and all charge states associated with a particular ion.

Percent glycation was quantified according to the formula

$$\%\;glycation=\frac{volume\;of\;AGE\;modified\;peptide}{total\;volume\;of\;\text{both}\;modified\;\text{and}\;unmodified\;peptide}$$

### Peptide Glycation Protocol

Peptide glycation reactions were carried out in Eppendorf tubes with a final volume of 50 μL. 10 mM MGO stocks were prepared by adding 15.38 μL commercial MGO solution (40% w/v) into 10 mL of ultrapure water and stored at 4 °C for up to 1 week. 100 mM PBS stocks (pH 7.3) were prepared using BupH Phosphate Buffer Saline packs according to instructions provided by Thermo Scientific (28372). Typically, a peptide in vitro glycation reaction contained 27.5 μL of ultrapure water, 10 μL of 10 mM MGO stock in water (2 mM final concentration), 10 μL of 100 mM PBS stock in water (20 mM final concentration), and 2.5 μL of 20 mM peptide stock solution in DMF (1 mM final concentration). Tubes were capped, briefly spun using a benchtop microcentrifuge, and incubated in a 37 °C water bath for up to 24 h or up to 6 weeks depending on the experiment. After incubation, reactions were diluted 100× with ultrapure water and 500 mM Tris buffer pH 7.4 (5 mM final concentration) to quench the reaction, and then subjected to LC–MS analysis.

### Peptide Glycation at Elevated Temperatures

Peptide glycation reactions were carried out according to the general glycation protocol for final concentrations. During screening, reactions were assembled in PCR tubes and incubated in a thermocycler (Bio-Rad) using a temperature gradient so that each temperature is set for each row (37 °C, 45 °C, 55 °C, 60 °C, 65 °C, and 70 °C). Subsequently, glycation reactions were performed at 37 and 60 °C with incubation in water baths.

### Peptide Glycation at Different pH

Peptide glycation reactions were carried out according to the general glycation protocol for final concentrations. 100 mM PBS stocks were prepared using BupH phosphate buffer saline packs according to instructions provided by Thermo Scientific (28372). The pH of each PBS stock was then adjusted with HCl (100 mM) or NaOH (100 mM) to the desired pH (7.3, 8.0, 9.0, 10.0, 10.2, 10.4, 10.6, 10.8, 11.0, and 12.00), monitoring with a benchtop pH meter (VWR).

### General Protocol for Dilution Experiments

For dilutions experiments described in main text Figure 2, peptides (1 mM) were incubated with MGO (2 mM) as described above. At time points of interest (typically 3 h and/or 24 h), an aliquot of the reaction mixture (10 μL) was diluted into 990 μL 20 mM PBS (pH 7.3, 100× dilution), briefly spun using a benchtop microcentrifuge, and further incubated in a 37 °C water bath for up to 48 additional hours. The resulting diluted reaction mixture was subjected to LC–MS analysis without any further dilution or purification.

### Mammalian Cell Culture and Sample Preparation of TMT-Labeled Peptides

150 mm culture dishes were seeded with 10 × 10^6^ HEK-293T cells in triplicates and grown to confluency in DMEM with 10% FBS supplementation. Cells were treated with 2 mM MGO in serum free DMEM for 24 h and harvested using TrypLE Express (Gibco). Cell pellets were lysed in 200 μL ice cold 8 M urea/50 mM triethylammonium bicarbonate (TEAB) with phosphatase/protease inhibitors (Pierce) and sonicated at 10% amplitude with 3 × 10 s pulses, followed by centrifugation at 15000 rpm for 15 min. Supernatant was collected, reduced with 8 mM dithiothreitol for 1 h, alkylated with 20 mM iodoacetamide for 30 min at room temperature in the dark. Subsequently, samples were digested overnight with trypsin at 37 °C. The digestion reaction was quenched using 0.1% trifluoroacetic acid, and samples were desalted using Pierce Peptide Desalting C_18_ Spin Columns (#89852). Peptides were collected and dried using a speedvac prior to TMT labeling.

### TMT Labeling Protocol

100 μg of the dried peptide samples were reconstituted in 100 μL of 100 mM TEAB buffer. Each sample was then labeled using TMT 10-plex as per the manufacturer’s instructions (TMT10plex Isobaric Label Reagents and Kits, #90111). The labeling reaction mixture was incubated at room temperature for 1 h and quenched using 5 μL of 5% hydroxylamine solution with incubation for 15 min at room temperature prior to pooling. The pooled sample was then dried, desalted and reconstituted in 0.1% formic acid for LC–MS/MS analysis. The 10 TMT channels are composed of three biological replicates (each with three technical replicates) and one pool sample, in which the sample ratio between the pool channel (i.e., channel 126) and channels 127N, 127C, 128N, 128C, 129N, 129C, 130N, 130C and 131 is expected to be 1. The experiment was carried out in two batches where the pooled samples (i.e., channel 126) served as the bridge channel. In the first batch, 3 biological replicates of the untreated samples were labeled with 127N, 127C, 128N TMT tags, 3 biological replicates of the samples treated with 0.5 mM MGO were labeled with 128C, 129N, 129C and 3 biological replicates of the samples treated with 1 mM MGO were labeled with 130N, 130C, 131 TMT tags. In the second batch, 3 biological replicates of the samples treated with 2 mM MGO were labeled with 127N, 127C, 128N TMT tags, 3 biological replicates of the untreated mitochondrial samples were labeled with 128C, 129N, 129C and 3 biological replicates of the mitochondrial samples treated with 1 mM MGO were labeled with 130N, 130C, 131 TMT tags.

### Mass Spectrometry Data Acquisition for Proteomic Analysis

LC–MS/MS data acquisition for TMT-labeled pooled sample was carried out using Thermo Scientific Orbitrap Exploris 240 (Thermo Fisher Scientific, Bremen, Germany) connected with Vanquish Neo UHPLC system (Thermo Fisher Scientific, Germany). Reconstituted peptides were loaded onto a trap column (300 μm × 5 mm, Thermo Scientific PepMap Neo Trap Cartridge, 174500). Peptides were separated on an analytical column (75 μM × 150 mm, Thermo Scientific EASY-Spray PepMap Neo UHPLC columns, ES75150PN) at a flow rate of 300 nL/min using an optimized linear gradient of 2–45% acetonitrile for 235 min. Mass spectrometry data were acquired in data-dependent acquisition (DDA) mode with “top speed” of 3 s cycle time. MS1 scans were acquired at a mass resolution of 60,000. The automatic gain control (AGC) target value for precursor ion acquisition was set to 1 × 10^6^ and maximum ion injection time was set to automatic mode. Scan range of masses was at 350–1400 *m*/*z*. RF lens was set at 70%. Isolation width of 0.4 *m*/*z* was used for precursor ion selection and fragmented using higher-energy collision dissociation (HCD) with 36% normalized collision energy. MS2 scans were acquired at a mass resolution of 45,000 with AGC target as 1 × 10^6^ and maximum ion injection time at auto mode. A FAIMS Pro Interface was connected to the Exploris 240 mass spectrometer. The compensation voltage (CV) of the FAIMS Pro interface was set to a combination of −45, −60 and −75 CVs. Each of the three CVs used was set to run DDA mode for 1 s cycle to build a big cycle of 3 s.

### Mass Spectrometry Proteomic Data Processing and Analysis

All RAW files were processed and analyzed using Thermo Proteome Discoverer (PD) v.3.0. All database searches were performed using MSFragger-PD node (PMID: 36475762) as the combination of MSFragger and PeptideProphet (Philosopher) processing nodes. The default settings were predominantly used in MSFragger-PD node unless otherwise stated. *Homo sapiens* (SwissProt TaxID 9606, 20422 proteins) was used for the protein database. For all searches, trypsin cleavage was set to full, a precursor mass tolerance of 20 ppm was used, with a fragment mass tolerance of 0.6 Da allowing tryptic peptides only with up to two missed cleavages. The raw data was searched for five dynamic PTM combinations that could be found on peptides. The static modifications included carbamidomethylation (+57.021 Da) of cysteine and TMT-modification (+229.163 Da) of lysine and peptide N-terminus, and the dynamic modifications included argpyrimidine (+80.026 Da, R), methylglyoxal-derived hydroxyimidazolone isomers (MGH-isomers) (+54.011 Da, R), carboxyethyl arginine/lysine or MGH-DH (CEA/CEL/MGH-DH) (+72.021, R, K), tetrahydropyriminidine (THP) (+144.041 Da, R), and phosphorylation (+79.966 Da; S, T, Y). A separate, parallel analysis was performed using the same static modifications but a different combination of dynamic modifications, including dimethylation (+28.031 Da, R), acetylation (+42.011 Da, R), argpyrimidine (+80.026 Da, R), and phosphorylation (+79.966 Da; S, T, Y). A “closed” search was performed to minimize the computational requirements for database searching for each technical replicate and all FAIMS parameters included in this study. PeptideProphet was used for PSM validation on these searches allowing a 1% FDR (false discovery rate). All bioinformatic analysis of LC–MS/MS data was performed in the R statistical computing environment. Gene Ontology (GO) enrichment analysis and functional annotation clustering analysis were performed using DAVID (PMID: 19131956) and proportional Venn diagrams were generated using https://www.deepvenn.com/. GO analysis for the APY gene list was also performed using ToppGene (http://toppgene.cchmc.org). The APY gene list was also manually inspected using UniProt ID Mapping tool (https://www.uniprot.org/id-mapping).

## Supplementary Material









## Notes

Additional experimental data, details, materials, and methods (PDF)
Supplementary Excel file 1: All AGE peptides from proteomic analysis of treated cells (XLSX)
Supplementary Excel file 2: DAVID GO analysis results, ToppGene GO analysis results for APY, DAVID functional clustering analysis results (XLSX)
Supplementary Excel file 3: UniProt ID mapping using APY gene IDs (XLSX)

## Abbreviations

AGE: advanced glycation end-product
APY: argpyrimidine
MGO: methylglyoxal
MGH-1, -2, & −3: hydroimidazolone isomers
MGH-DH: dihydroxyimidazolidine
CEA: carboxyethylarginine
THP: tetrahydropyrimidine
LC–MS: liquid chromatography–mass spectrometry
HPLC: high-performance liquid chromatography
TMT: tandem mass tag
GO analysis: gene ontology analysis
